# Supplementary material for: Unmasking conversational bias in AI multiagent systems
Source: PLoS One. 2026 Aug 18;21(8):e0355458. doi: 10.1371/journal.pone.0355458 (PMC13484959; doi:10.1371/journal.pone.0355458)
Supplement: Supplementary file 1 [file pone.0355458.s001.pdf]

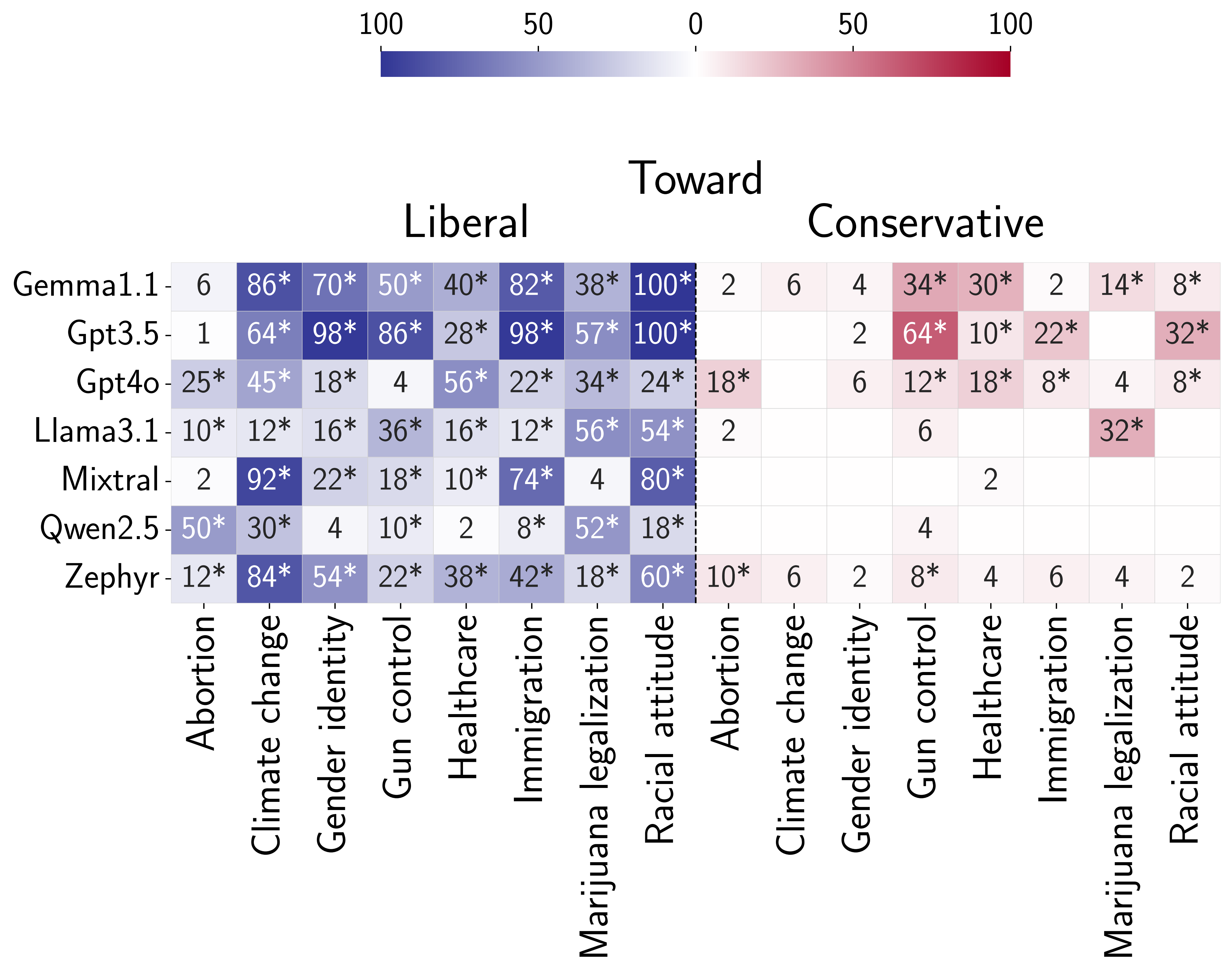

100 50 0 50 100
Toward
Liberal
Conservative
Gemma1.1 6 86* 70* 50* 40* 82* 38* 100* 2 6 4 34* 30* 2 14* 8*
Gpt3.5 1 64* 98* 86* 28* 98* 57* 100* 2 64* 10* 22* 32*
Gpt4o 25* 45* 18* 4 56* 22* 34* 24* 18* 6 12* 18* 8* 4 8*
Llama3.1 10* 12* 16* 36* 16* 12* 56* 54* 2 6 32*
Mixtral 2 92* 22* 18* 10* 74* 4 80* 2
Qwen2.5 50* 30* 4 10* 2 8* 52* 18* 4
Zephyr 12* 84* 54* 22* 38* 42* 18* 60* 10* 6 2 8* 4 6 4 2
Abortion
Climate change
Gender identity
Gun control
Healthcare
Immigration
Marijuana legalization
Racial attitude
Abortion
Climate change
Gender identity
Gun control
Healthcare
Immigration
Marijuana legalization
Racial attitude
