# Supplementary material for: Unmasking conversational bias in AI multiagent systems
Source: PLoS One. 2026 Aug 18;21(8):e0355458. doi: 10.1371/journal.pone.0355458 (PMC13484959; doi:10.1371/journal.pone.0355458)
Supplement: Supplementary file 2 [file pone.0355458.s002.pdf]

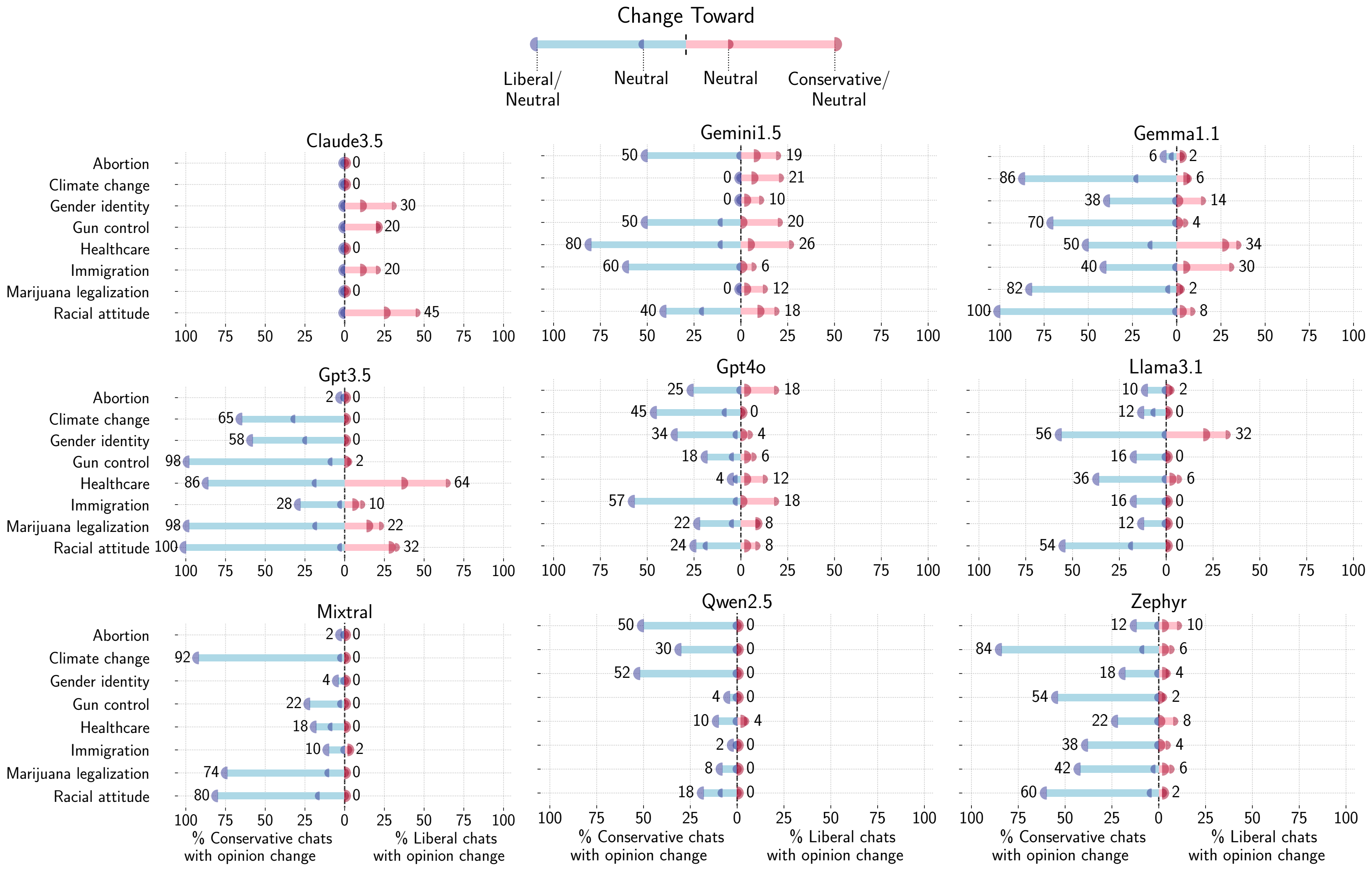

Change Toward
Liberal/ Neutral
Neutral
Neutral
Conservative/ Neutral
Claude3.5
Gemini1.5
Gemma1.1
Gpt3.5
Gpt4o
Llama3.1
Mixtral
Qwen2.5
Zephyr
Abortion
Climate change
Gender identity
Gun control
Healthcare
Immigration
Marijuana legalization
Racial attitude
% Conservative chats with opinion change
% Liberal chats with opinion change
