# Supplementary material for: Unmasking conversational bias in AI multiagent systems
Source: PLoS One. 2026 Aug 18;21(8):e0355458. doi: 10.1371/journal.pone.0355458 (PMC13484959; doi:10.1371/journal.pone.0355458)
Supplement: Supplementary file 3 [file pone.0355458.s003.pdf]

	Toward Liberal: 2 of 2	Toward Liberal: 1 of 2	Toward Conservative: 1 of 2	Toward Conservative: 2 or 2
Abortion	-	-		
Climate change	-	-		
Gender identity	-	-		
Gun control	-	-	6	
Healthcare	-	-		4
Immigration	-	-		
Marijuana legalization	-	-	4	
Racial attitude	-	-	12	6

	Toward Liberal: 2 of 2	Toward Liberal: 1 of 2	Toward Conservative: 1 of 2	Toward Conservative: 2 or 2
Abortion		50	30	
Climate change			10	20
Gender identity			10	
Gun control	10	40	20	
Healthcare	10	70	10	10
Immigration		60		
Marijuana legalization				
Racial attitude	10	30	20	

	Toward Liberal: 2 of 2	Toward Liberal: 1 of 2	Toward Conservative: 1 of 2	Toward Conservative: 2 or 2
Abortion		6	2	
Climate change	62	24	6	
Gender identity	28	10	14	
Gun control	58	12	4	
Healthcare	32	18	24	10
Immigration	18	22	16	14
Marijuana legalization	52	30	2	
Racial attitude	94	6	6	2

	2 of 2	1 of 2	1 of 2	2 or 2
Abortion		2		
Climate change	38	28		
Gender identity	2	56		
Gun control	76	22	2	
Healthcare	42	44	50	14
Immigration		28	20	
Marijuana legalization	74	26	16	6
Racial attitude	90	10	28	4

	2 of 2	1 of 2	1 of 2	2 or 2
Abortion	4	22	16	2
Climate change	10	36		
Gender identity	12	22	2	2
Gun control		18	6	
Healthcare		4	12	
Immigration	4	54	14	4
Marijuana legalization	4	18	8	
Racial attitude	6	18	4	4

	2 of 2	1 of 2	1 of 2	2 or 2
Abortion	2	8	2	
Climate change		12		
Gender identity	2	54	30	2
Gun control		16		
Healthcare	6	30	4	2
Immigration	2	14		
Marijuana legalization	4	8		
Racial attitude	18	36		

	2 of 2	1 of 2	1 of 2	2 or 2
Abortion		2		
Climate change	78	14		
Gender identity		4		
Gun control	6	16		
Healthcare	4	14		
Immigration	4	6	2	
Marijuana legalization	48	26		
Racial attitude	40	40		

	2 of 2	1 of 2	1 of 2	2 or 2
Abortion	16	34		
Climate change		30		
Gender identity	6	46		
Gun control		4		
Healthcare		10	4	
Immigration		2		
Marijuana legalization	2	6		
Racial attitude	6	12		

	2 of 2	1 of 2	1 of 2	2 or 2
Abortion	4	8	8	2
Climate change	68	16	4	2
Gender identity	8	10	2	2
Gun control	32	22	2	
Healthcare	8	14	6	2
Immigration	20	18	4	
Marijuana legalization	18	24	6	
Racial attitude	36	24	2	
