# Supplementary material for: Unmasking conversational bias in AI multiagent systems
Source: PLoS One. 2026 Aug 18;21(8):e0355458. doi: 10.1371/journal.pone.0355458 (PMC13484959; doi:10.1371/journal.pone.0355458)
Supplement: Supplementary file 4 [file pone.0355458.s004.pdf]

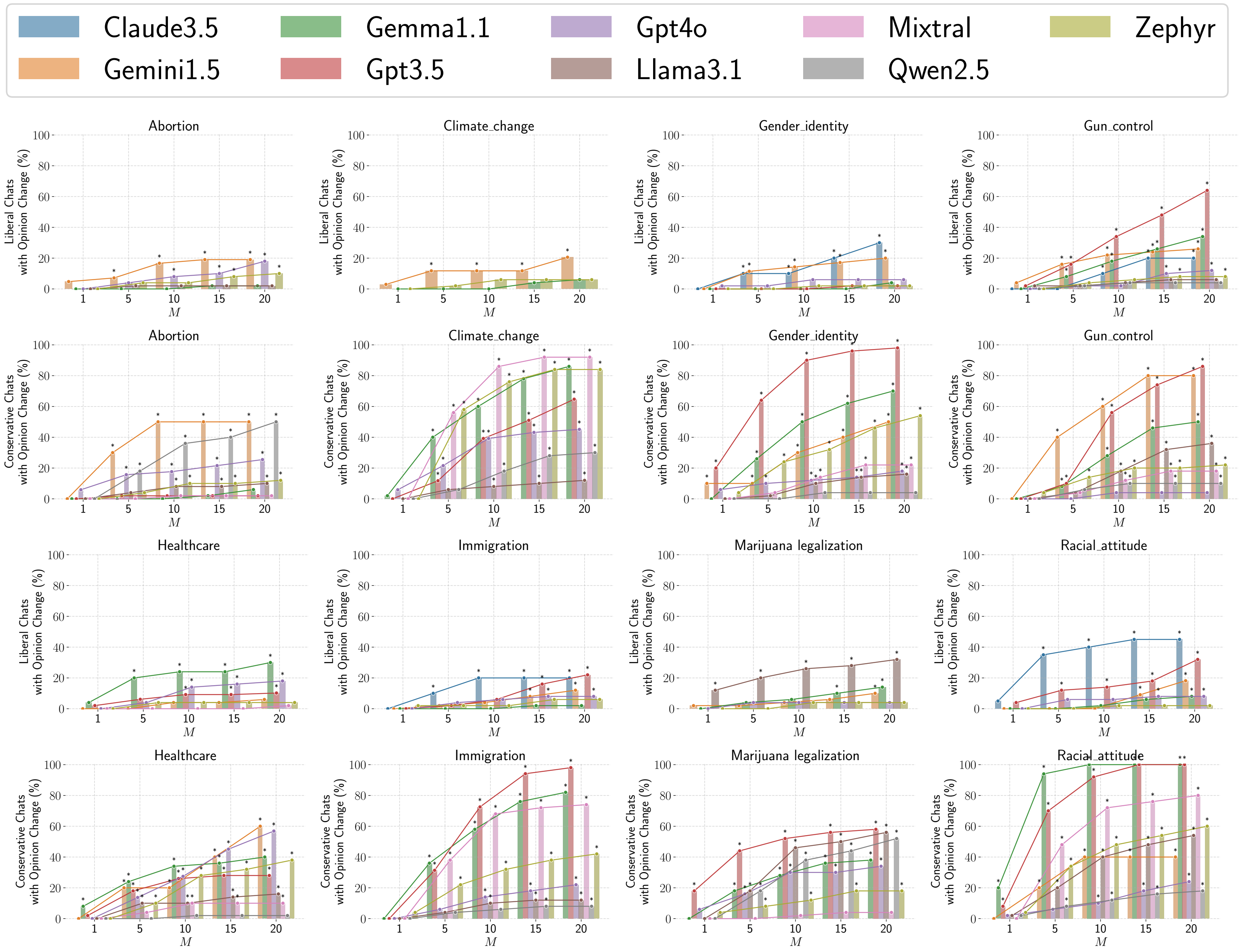

Claude3.5
Gemma1.1
Gpt4o
Mixtral
Zephyr
Gemini1.5
Gpt3.5
Llama3.1
Qwen2.5
Abortion
Climate_change
Gender_identity
Gun_control
Healthcare
Immigration
Marijuana legalization
Racial_attitude
Liberal Chats with Opinion Change (%)
Conservative Chats with Opinion Change (%)
M
