# Supplementary material for: Unmasking conversational bias in AI multiagent systems
Source: PLoS One. 2026 Aug 18;21(8):e0355458. doi: 10.1371/journal.pone.0355458 (PMC13484959; doi:10.1371/journal.pone.0355458)
Supplement: Supplementary file 5 [file pone.0355458.s005.pdf]

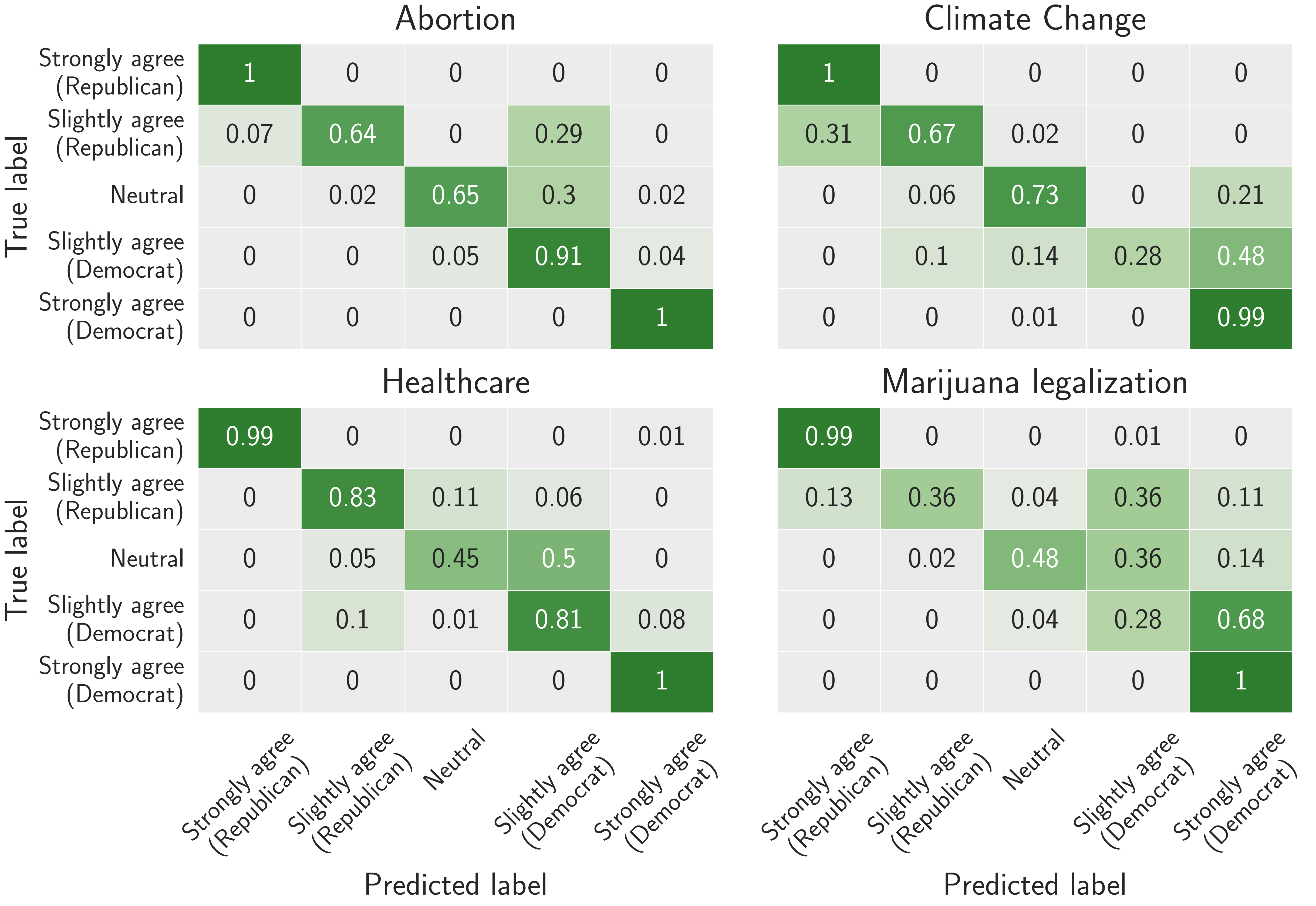
Abortion
Climate Change
Healthcare
Marijuana legalization
True label
Predicted label
Strongly agree (Republican)
Slightly agree (Republican)
Neutral
Slightly agree (Democrat)
Strongly agree (Democrat)
Abortion: 1 0 0 0 0 / 0.07 0.64 0 0.29 0 / 0 0.02 0.65 0.3 0.02 / 0 0 0.05 0.91 0.04 / 0 0 0 0 1
Climate Change: 1 0 0 0 0 / 0.31 0.67 0.02 0 0 / 0 0.06 0.73 0 0.21 / 0 0.1 0.14 0.28 0.48 / 0 0 0.01 0 0.99
Healthcare: 0.99 0 0 0 0.01 / 0 0.83 0.11 0.06 0 / 0 0.05 0.45 0.5 0 / 0 0.1 0.01 0.81 0.08 / 0 0 0 0 1
Marijuana legalization: 0.99 0 0 0.01 0 / 0.13 0.36 0.04 0.36 0.11 / 0 0.02 0.48 0.36 0.14 / 0 0 0.04 0.28 0.68 / 0 0 0 0 1
