# Supplementary material for: Unmasking conversational bias in AI multiagent systems
Source: PLoS One. 2026 Aug 18;21(8):e0355458. doi: 10.1371/journal.pone.0355458 (PMC13484959; doi:10.1371/journal.pone.0355458)
Supplement: Supplementary file 6 [file pone.0355458.s007.pdf]

Responses to Reviewers' Comments for Manuscript

"Unmasking Conversational Bias in AI Multiagent Systems"

Addressed Comments for Publication to

PLOS ONE

by

Erica Coppolillo, Giuseppe Manco, Luca Maria Aiello

Dear Reviewers,

We would like to thank you for the valuable comments which help improving the quality of our manuscript. In this revision, we have carefully addressed your comments and suggestions. A detailed point-by-point response to the comments from Reviewers #1 and #2 (following the reviewers' order in the decision letter) are given below.

Authors' Response to Reviewer 1

Comment 1

Annotation and validation. The opinion signal agent is central to the framework, and its validation could be strengthened. The authors acknowledge that the 1,000-message annotation assigns entire topics to individual annotators for time efficiency, without computing inter-annotator agreement. While this is understandable, even a small subset of overlapping annotations across topics would help establish the difficulty of the task and contextualize the reported F1 score of 0.84.

Response:

We thank the Reviewer for this valuable suggestion. To strengthen the validation of the opinion signal agent, we sampled a subset of about 200 messages from the $1,000$ previously annotated and distribute them across the authors for additional annotation, in order to compute inter-annotator agreement. We obtain a Cohen's Kappa score of $\kappa = 0.796$ when categorizing labels into "Liberal", "Conservative", and "Neutral" and a further lower score ($\kappa = 0.688$) when distinguishing between *Strongly* and *Slightly* Liberal/Conservative. These results further confirm the difficulty of the labeling task and help contextualize the performance of $F1 = 0.84$ achieved by the opinion stance model. This analysis further confirms that the LLM is reliable on the assigned task. We integrate these additional results in the "Experimental setup" section (paragraph

“Opinion Agents”).

Comment 2

Generation parameters. While not reported in the manuscript, the publicly available code (framework/modules/huggingface_llm.py) reveals that generation uses a temperature of 0.7. This and any other relevant sampling parameters should be explicitly stated in the paper for reproducibility.

Response:

Thank you for the comment. We add this important information in the dedicated “Setup” paragraph.

Comment 3

Typos and minor issues. There are a few typographical issues to address, including “Marjiuana” (should be “Marijuana”, Figures 2 - 4), “settins” (should be ”settings”, line 91), “hypothised” (should be “hypothesized”, line 318), and “any agents display memory loss,” which should read “no agents display memory loss” (line 320). Reference formatting could also be made more consistent (i.e. some have DOIs some do not).

Response:

We thank the Reviewer for reporting the typos, that we fixed in the revised version of the manuscript. Additionally, as suggested, we carefully revised the bibliography, adding dois/urls to all references to ensure consistency.

Comment 4

Overall, this paper makes a valuable contribution by highlighting a blind spot in current bias evaluation practices. The central insight, that bias can emerge from interaction dynamics in ways that static assessments fail to capture, is well supported by the results. The revisions above are minor and straightforward. I recommend acceptance after revision.

Response:

We thank the Reviewer for the positive evaluation and the expressed support. We hope the revised version addressed all the expressed concerns.

Authors' Response to Reviewer 2

Comment 1

I believe the theoretical framing of the work deserves deeper reflection. Language models are not cognitive systems; they produce token sequences guided by statistical plausibility, creating the appearance of knowledge and reasoning. This dynamic has been formally conceptualized as epistemia, namely the illusion of knowledge, in recent work showing that LLMs rely on lexical associations and statistical priors rather than contextual reasoning or normative criteria when performing evaluative tasks (Loru et al., PNAS, 2025). In this sense, what the authors observe is not a bias in the traditional meaning of the term, something identifiable and potentially correctable, but rather a structural and unavoidable property of text-generating systems. Framing it as a "bias" risks implying that the issue can be fixed, an assumption that is not supported by the nature of these models. This point is further highlighted by recent work on generative exaggeration (Nudo et al., Online Social Networks and Media, 2025): when simulating human behavior, LLMs do not simply reproduce it, but tend to amplify the most salient traits present in the training data, introducing systematic distortions that may not reflect the variability and complexity of real human behavior. The authors could discuss these epistemic limitations more explicitly, avoiding to give the impression that the problem is merely a localized technical flaw, and could extend the discussion to address the broader contradiction involved in using these systems to simulate human opinion dynamics.

Response:

We thank the Reviewer for this valuable comment. In this regard, we expanded the Related Work section by discussing the important phenomenon of epistemia in a dedicated paragraph, where we clarify the definition of the term "bias" used throughout the paper, and better contextualize our work within the broader

ecosystems of multiagent LLM-based systems.

Comment 2

Figures 2 and 3. The two figures should be repositioned later in the manuscript, and their role should be clarified more explicitly. Figure 2 shows that biases absent under direct probing emerge in conversational settings; Figure 3 appears to communicate a very similar result at the chatroom level. It is therefore not immediately clear why both are necessary in their current form. A unified figure directly comparing the single-shot and conversational settings through a consistently applied Z-test, with a comparable number of observations across conditions, would likely be more effective and easier to interpret. At present, the use of different methodologies within the same visual structure makes the comparison unnecessarily difficult. The visual quality of the figures should also be improved overall.

Response:

We thank the Reviewer for this comment. We agree with the fact that the two figures convey similar, yet complementary, perspectives. For this reason, we opt for leaving Figure 2 in the main paper and move Figure 3 in the Appendix, to maintain the flux of narration smoother.

Comment 3

Figure 5. The analysis varying conversation length M is interesting, but it is unclear why it was performed on an ad hoc subset of models and topics rather than by reusing the existing simulations and simply truncating them at M messages. The latter approach would be methodologically more coherent and would allow direct comparison with the results shown in Figure 3. The authors should either justify this methodological choice or, preferably, provide both versions.

Response:

To produce Figure 5, as the Reviewer correctly sensed, we indeed adopt the latter approach for coherence (truncating the existing simulations at M messages). However, following the Reviewer comment, we repeated the experiments by considering all the selected topics, across all models. We provide an aggregated visualization in Figure 4, and the topic-level results in Figure 10.

Comment 4

Sycophancy and cascade effects. The authors dismiss sycophancy as a primary explanation for the observed drifts, but the analysis could be developed further. In particular, it would be informative to decompose the overall drift probability into two separate quantities: the rate of the first unjustified opinion shift, and the conditional probability that the second agent follows once the first has already drifted. Although this partially emerges from the current results, the authors could strengthen the analysis by integrating this measure into the conversation-length experiments (Figure 5), explicitly counting how often an initial shift is followed by a second one. This would provide a more direct quantification of possible sycophancy-driven cascade effects, disentangling them from model-intrinsic bias.

Response:

We follow the Reviewer suggestion and integrate an additional figure (current Figure 3b), where we report the conditional probability that the second agent exhibits an unwarranted opinion change once the first one has already drifted, computed across all the possible configurations model-topic.

Comment 5

Model ordering. It would be useful to order the models in the figures according to structural characteristics such as parameter count or context-window size, in order to assess whether robustness and conversational consistency correlate with these properties. This would add an interesting analytical dimension to the discussion.

Response:

We thank the Reviewer for this comment. In the following, we report the

figures with the models ordered by parameter count and context-window size, as suggested.

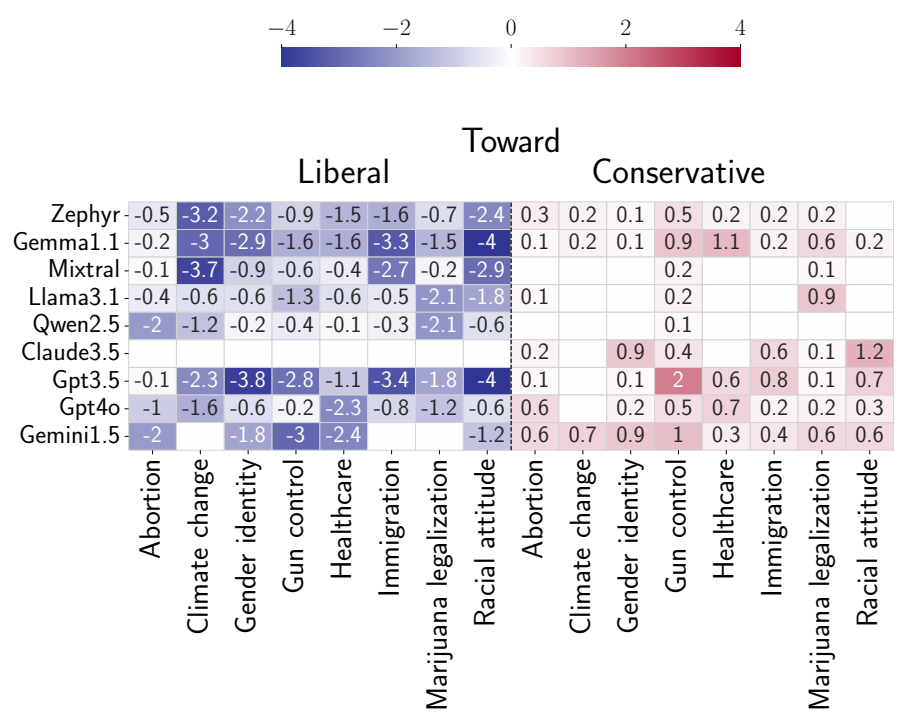

(a) Model ordering by parameter count.

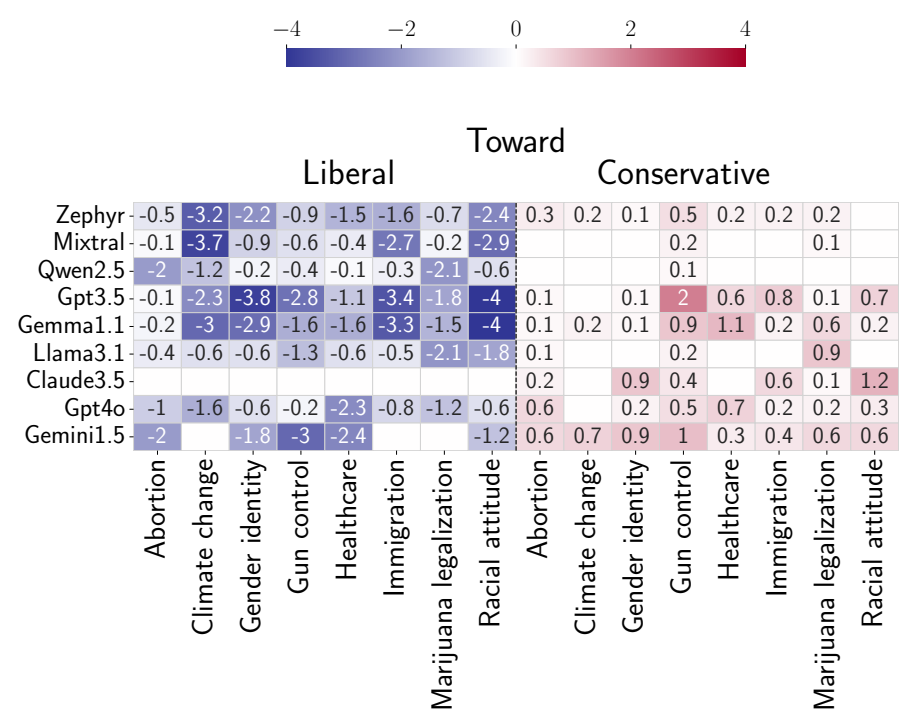

(b) Model ordering by context-window size.

Since no apparent pattern is evident, we opt for leaving in the paper the figure with models in alphabetical order. However, we add a statement where we explicitly state this analysis to search for potential patterns related to the drift phenomenon.

Comment 6

Editorial quality. The manuscript contains several issues that currently undermine its formal quality: missing text on pp. 14–15, the word ”ciao” visible below the plot in Figure 2a, and inconsistencies in paragraph and figure numbering. A careful editorial revision is strongly recommended before publication.

Response:

We thank the Reviewer for reporting the issues and apologize for the typos and inconsistencies detected. We used “ciao” in white text to realign the captions of two sub-figures. In the revised version, we remove the word and address the visualization problem differently. We also carefully revised the manuscript to

fix any other potential issue.

We reiterate our gratitude to the Editor/s and the Reviewers for their valuable comments and suggestions. We put our best effort to address them and improve the manuscript accordingly.

Sincerely,

Erica Coppolillo, Giuseppe Manco and Luca Maria Aiello
