# Supplementary material for: Unmasking conversational bias in AI multiagent systems
Source: PLoS One. 2026 Aug 18;21(8):e0355458. doi: 10.1371/journal.pone.0355458 (PMC13484959; doi:10.1371/journal.pone.0355458)
Supplement: Peer review history

# Peer review: Decision Letter 0

**DOI:** 10.1371/journal.pone.0355458.r001

**Authors:** Alessandro Galeazzi

20 May 2026

PONE-D-26-14441

Unmasking Conversational Bias in AI Multiagent Systems

PLOS One

Dear Dr. Coppolillo,

Thank you for submitting your manuscript to PLOS ONE. After careful consideration, we feel that it has merit but does not fully meet PLOS ONE’s publication criteria as it currently stands. Therefore, we invite you to submit a revised version of the manuscript that addresses the points raised during the review process.

==============================

Your manuscript has been reviewed by two experts, who found great value in the work. However, Reviewer 1 highlighted a lack of certain technical details, while Reviewer 2 pointed out potential ambiguity in the framing and suggested minor organizational changes. I agree with their comments and strongly believe that a revision will significantly improve the manuscript. I encourage the authors to carefully address each point raised by the reviewers.

==============================

Please submit your revised manuscript by Jul 04 2026 11:59PM. If you will need more time than this to complete your revisions, please reply to this message or contact the journal office at plosone@plos.org. When you're ready to submit your revision, log on to https://www.editorialmanager.com/pone/ and select the 'Submissions Needing Revision' folder to locate your manuscript file.

Please include the following items when submitting your revised manuscript:

- A letter that responds to each point raised by the academic editor and reviewer(s). You should upload this letter as a separate file labeled 'Response to Reviewers'.
- A marked-up copy of your manuscript that highlights changes made to the original version. You should upload this as a separate file labeled 'Revised Manuscript with Track Changes'.
- An unmarked version of your revised paper without tracked changes. You should upload this as a separate file labeled 'Manuscript'.

If you would like to make changes to your financial disclosure, please include your updated statement in your cover letter. Guidelines for resubmitting your figure files are available below the reviewer comments at the end of this letter.

If applicable, we recommend that you deposit your laboratory protocols in protocols.io to enhance the reproducibility of your results. Protocols.io assigns your protocol its own identifier (DOI) so that it can be cited independently in the future. For instructions see: https://journals.plos.org/plosone/s/submission-guidelines#loc-laboratory-protocols. Additionally, PLOS ONE offers an option for publishing peer-reviewed Lab Protocol articles, which describe protocols hosted on protocols.io. Read more information on sharing protocols at https://plos.org/protocols?utm_medium=editorial-email&utm_source=authorletters&utm_campaign=protocols.

As the corresponding author, your ORCID iD is verified in the submission system and will appear in the published article. PLOS supports the use of ORCID, and we encourage all coauthors to register for an ORCID iD and use it as well. Please encourage your coauthors to verify their ORCID iD within the submission system before final acceptance, as unverified ORCID iDs will not appear in the published article. *Only*  the individual author can complete the verification step; PLOS staff *cannot*  verify ORCID iDs on behalf of authors.

We look forward to receiving your revised manuscript.

Kind regards,

Alessandro Galeazzi, PhD

Academic Editor

PLOS One

**Journal Requirements:**

1. When submitting your revision, we need you to address these additional requirements.

Please ensure that your manuscript meets PLOS ONE's style requirements, including those for file naming. The PLOS ONE style templates can be found at

https://journals.plos.org/plosone/s/file?id=wjVg/PLOSOne_formatting_sample_main_body.pdf and

https://journals.plos.org/plosone/s/file?id=ba62/PLOSOne_formatting_sample_title_authors_affiliations.pdf

2. Thank you for stating the following financial disclosure:

LMA acknowledges the support from the Carlsberg Foundation through the

COCOONS project (CF21-0432).

EC acknowledges the partial support from  MUR on D.M. 351/2022, PNRR Ricerca, CUP H23C22000440007.

Please state what role the funders took in the study.  If the funders had no role, please state: "The funders had no role in study design, data collection and analysis, decision to publish, or preparation of the manuscript."

If this statement is not correct you must amend it as needed.

Please include this amended Role of Funder statement in your cover letter; we will change the online submission form on your behalf.

3. Thank you for stating the following in the Acknowledgments Section of your manuscript:

We thank Simon Martin Breum, Daniel Vædele Egdal, Victor Gram Mortensen, and Anders Giovanni Møller for providing the computational framework to simulate agent conversations, and for the early research insights of their work that motivated our contribution. LMA acknowledges the support from the Carlsberg Foundation through the COCOONS project (CF21-0432). This work has also been partially funded by MUR on D.M. 351/2022, PNRR Ricerca, CUP H23C22000440007.

We note that you have provided funding information that is not currently declared in your Funding Statement. However, funding information should not appear in the Acknowledgments section or other areas of your manuscript. We will only publish funding information present in the Funding Statement section of the online submission form.

Please remove any funding-related text from the manuscript and let us know how you would like to update your Funding Statement. Currently, your Funding Statement reads as follows:

LMA acknowledges the support from the Carlsberg Foundation through the

COCOONS project (CF21-0432).

EC acknowledges the partial support from  MUR on D.M. 351/2022, PNRR Ricerca, CUP H23C22000440007.

Please include your amended statements within your cover letter; we will change the online submission form on your behalf.

4. Please note that your Data Availability Statement is currently missing the direct link to access each database. If your manuscript is accepted for publication, you will be asked to provide these details on a very short timeline. We therefore suggest that you provide this information now, though we will not hold up the peer review process if you are unable.

5. Please upload a new copy of Figures 2, 3, 8, 9 and 10, as the detail is not clear. Please follow the link for more information:  https://journals.plos.org/plosone/s/figures

6. If the reviewer comments include a recommendation to cite specific previously published works, please review and evaluate these publications to determine whether they are relevant and should be cited. There is no requirement to cite these works unless the editor has indicated otherwise.

[Note: HTML markup is below. Please do not edit.]

Reviewers' comments:

Reviewer's Responses to Questions

**Comments to the Author**

1. Is the manuscript technically sound, and do the data support the conclusions?

The manuscript must describe a technically sound piece of scientific research with data that supports the conclusions. Experiments must have been conducted rigorously, with appropriate controls, replication, and sample sizes. The conclusions must be drawn appropriately based on the data presented.

Reviewer #1: Yes

Reviewer #2: Yes

**********

2. Has the statistical analysis been performed appropriately and rigorously?

Reviewer #1: Yes

Reviewer #2: Yes

**********

3. Have the authors made all data underlying the findings in their manuscript fully available?

The PLOS Data policy requires authors to make all data underlying the findings described in their manuscript fully available without restriction, with rare exception (please refer to the Data Availability Statement in the manuscript PDF file). The data should be provided as part of the manuscript or its supporting information, or deposited to a public repository. For example, in addition to summary statistics, the data points behind means, medians and variance measures should be available. If there are restrictions on publicly sharing data—e.g. participant privacy or use of data from a third party—those must be specified.

Reviewer #1: Yes

Reviewer #2: Yes

**********

4. Is the manuscript presented in an intelligible fashion and written in standard English?

PLOS ONE does not copyedit accepted manuscripts, so the language in submitted articles must be clear, correct, and unambiguous. Any typographical or grammatical errors should be corrected at revision, so please note any specific errors here.

Reviewer #1: Yes

Reviewer #2: Yes

**********

5. Review Comments to the Author

Please use the space provided to explain your answers to the questions above. You may also include additional comments for the author, including concerns about dual publication, research ethics, or publication ethics. (Please upload your review as an attachment if it exceeds 20,000 characters)

**Reviewer #1:**  Dear Editor,

Thank you for the opportunity to review this manuscript.

This paper addresses an important question: how biases in large language models manifest and evolve during multi-agent interactions. The authors introduce a framework simulating echo chamber conversations between LLM-powered agents and show that substantial opinion drift, undetected by standard questionnaire-based bias assessments, can emerge. This is both novel and practically relevant.

The paper has several strengths. The experimental design is clear and well-motivated, with the echo chamber setup providing a natural baseline (zero opinion shift). The evaluation is comprehensive, covering nine models, eight topics, and multiple sensitivity analyses (e.g., conversation length, prompt ablation, number of agents, memory saturation, and alternative opinion signals). The comparison between direct probing (Figure 2a) and conversational framework (Figure 2b) is particularly compelling, highlighting a key limitation of current bias evaluation methods. The open code and candid discussion of limitations are also appreciated.

I have a few suggestions that I believe would further strengthen the manuscript:

1. Annotation and validation. The opinion signal agent is central to the framework, and its validation could be strengthened. The authors acknowledge that the 1,000-message annotation assigns entire topics to individual annotators for time efficiency, without computing inter-annotator agreement. While this is understandable, even a small subset of overlapping annotations across topics would help establish the difficulty of the task and contextualize the reported F1 score of 0.84.

2. Generation parameters. While not reported in the manuscript, the publicly available code (framework/modules/huggingface_llm.py) reveals that generation uses a temperature of 0.7. This and any other relevant sampling parameters should be explicitly stated in the paper for reproducibility.

3. Typos and minor issues. There are a few typographical issues to address, including “Marjiuana” (should be “Marijuana”, Figures 2 - 4), “settins” (should be "settings", line 91), “hypothised” (should be “hypothesized”, line 318), and “any agents display memory loss,” which should read “no agents display memory loss” (line 320). Reference formatting could also be made more consistent (i.e. some have DOIs some do not).

Overall, this paper makes a valuable contribution by highlighting a blind spot in current bias evaluation practices. The central insight, that bias can emerge from interaction dynamics in ways that static assessments fail to capture, is well supported by the results. The revisions above are minor and straightforward. I recommend acceptance after revision.

**Reviewer #2:**  This paper addresses a timely and methodologically relevant topic, highlighting a fundamental inconsistency in the use of Large Language Models within opinion dynamics models. The core contribution, namely showing that biases absent under direct probing emerge strongly in conversational settings, is solid and well executed.

However, I believe the theoretical framing of the work deserves deeper reflection. Language models are not cognitive systems; they produce token sequences guided by statistical plausibility, creating the appearance of knowledge and reasoning. This dynamic has been formally conceptualized as epistemia, namely the illusion of knowledge, in recent work showing that LLMs rely on lexical associations and statistical priors rather than contextual reasoning or normative criteria when performing evaluative tasks (Loru et al., PNAS, 2025). In this sense, what the authors observe is not a bias in the traditional meaning of the term, something identifiable and potentially correctable, but rather a structural and unavoidable property of text-generating systems. Framing it as a "bias" risks implying that the issue can be fixed, an assumption that is not supported by the nature of these models. This point is further highlighted by recent work on generative exaggeration (Nudo et al., Online Social Networks and Media, 2025): when simulating human behavior, LLMs do not simply reproduce it, but tend to amplify the most salient traits present in the training data, introducing systematic distortions that may not reflect the variability and complexity of real human behavior. The authors could discuss these epistemic limitations more explicitly, avoiding to give the impression that the problem is merely a localized technical flaw, and could extend the discussion to address the broader contradiction involved in using these systems to simulate human opinion dynamics.

Furthermore other points to consider are:

1. Figures 2 and 3. The two figures should be repositioned later in the manuscript, and their role should be clarified more explicitly. Figure 2 shows that biases absent under direct probing emerge in conversational settings; Figure 3 appears to communicate a very similar result at the chatroom level. It is therefore not immediately clear why both are necessary in their current form. A unified figure directly comparing the single-shot and conversational settings through a consistently applied Z-test, with a comparable number of observations across conditions, would likely be more effective and easier to interpret. At present, the use of different methodologies within the same visual structure makes the comparison unnecessarily difficult. The visual quality of the figures should also be improved overall.

2. Figure 5. The analysis varying conversation length M is interesting, but it is unclear why it was performed on an ad hoc subset of models and topics rather than by reusing the existing simulations and simply truncating them at M messages. The latter approach would be methodologically more coherent and would allow direct comparison with the results shown in Figure 3. The authors should either justify this methodological choice or, preferably, provide both versions.

3. Sycophancy and cascade effects. The authors dismiss sycophancy as a primary explanation for the observed drifts, but the analysis could be developed further. In particular, it would be informative to decompose the overall drift probability into two separate quantities: the rate of the first unjustified opinion shift, and the conditional probability that the second agent follows once the first has already drifted. Although this partially emerges from the current results, the authors could strengthen the analysis by integrating this measure into the conversation-length experiments (Figure 5), explicitly counting how often an initial shift is followed by a second one. This would provide a more direct quantification of possible sycophancy-driven cascade effects, disentangling them from model-intrinsic bias.

4. Model ordering. It would be useful to order the models in the figures according to structural characteristics such as parameter count or context-window size, in order to assess whether robustness and conversational consistency correlate with these properties. This would add an interesting analytical dimension to the discussion.

5. Editorial quality. The manuscript contains several issues that currently undermine its formal quality: missing text on pp. 14–15, the word "ciao" visible below the plot in Figure 2a, and inconsistencies in paragraph and figure numbering. A careful editorial revision is strongly recommended before publication.

Loru E., Nudo J., Di Marco N., Santirocchi A., Atzeni R., Cinelli M., Cestari V., Rossi-Arnaud C., Quattrociocchi W. (2025). *The simulation of judgment in LLMs*. PNAS, 122(42), e2518443122.

Nudo J., Pandolfo M.E., Loru E., Samory M., Cinelli M., Quattrociocchi W. (2025). *Generative Exaggeration in LLM Social Agents: Consistency, Bias, and Toxicity*. Online Social Networks and Media.

**********

6. PLOS authors have the option to publish the peer review history of their article (what does this mean?). If published, this will include your full peer review and any attached files.

If you choose “no”, your identity will remain anonymous but your review may still be made public.

**Do you want your identity to be public for this peer review?**  For information about this choice, including consent withdrawal, please see our Privacy Policy.

Reviewer #1: No

Reviewer #2: No

**********

[NOTE: If reviewer comments were submitted as an attachment file, they will be attached to this email and accessible via the submission site. Please log into your account, locate the manuscript record, and check for the action link "View Attachments". If this link does not appear, there are no attachment files.]

To ensure your figures meet our technical requirements, please review our figure guidelines: https://journals.plos.org/plosone/s/figures

You may also use PLOS’s free figure tool, NAAS, to help you prepare publication quality figures: https://journals.plos.org/plosone/s/figures#loc-tools-for-figure-preparation.

NAAS will assess whether your figures meet our technical requirements by comparing each figure against our figure specifications.

# Author comment: Author response to Decision Letter 1

**DOI:** 10.1371/journal.pone.0355458.r002

30 Jun 2026

We sincerely thank the Editor and Reviewers for their valuable feedback, which has helped us refine the robustness of our work, in terms of both experimental evaluation and presentation quality.

A summary of the key changes is as follows:

1. We broaden the experimental section by: (i) strengthening the validation of the opinion signal model by further sampling a subset of about $200$ messages and distribute them across all authors to compute inter-annotator agreement; and (ii) computing the conditional probability that a second agent exhibits an unwarranted opinion change once the first has previously drifted.

2. We expanded the Related Work section to discuss the crucial phenomenon of epistemia, and better contextualize our work within the broad landscape of LLM-based systems.

3. We carefully revised the bibliography, ensuring consistency across references and adding dois/urls.

4. Overall, we improved the presentation quality of the paper, by fixing the reported typos, providing new figures to further corroborate the results, and enlarging the existing ones to enhance readability.

The complete point-by-point responses are provided in the revised cover letter.

Attachment Submitted filename: reviewer_responses.pdf

# Peer review: Decision Letter 1

**DOI:** 10.1371/journal.pone.0355458.r003

**Authors:** Alessandro Galeazzi

22 Jul 2026

Unmasking Conversational Bias in AI Multiagent Systems

PONE-D-26-14441R1

Dear Dr. Coppolillo,

We’re pleased to inform you that your manuscript has been judged scientifically suitable for publication and will be formally accepted for publication once it meets all outstanding technical requirements.

Within one week, you’ll receive an e-mail detailing the required amendments. When these have been addressed, you’ll receive a formal acceptance letter and your manuscript will be scheduled for publication.

An invoice will be generated when your article is formally accepted. Please note, if your institution has a publishing partnership with PLOS and your article meets the relevant criteria, all or part of your publication costs will be covered. Please make sure your user information is up-to-date by logging into Editorial Manager at Editorial Manager® and clicking the ‘Update My Information' link at the top of the page. For questions related to billing, please contact billing support.

If your institution or institutions have a press office, please notify them about your upcoming paper to help maximize its impact. If they’ll be preparing press materials, please inform our press team as soon as possible -- no later than 48 hours after receiving the formal acceptance. Your manuscript will remain under strict press embargo until 2 pm Eastern Time on the date of publication. For more information, please contact onepress@plos.org.

Kind regards,

Alessandro Galeazzi, PhD

Academic Editor

PLOS One

Additional Editor Comments (optional):

Both reviewers found the manuscript improved and ready for publications. I agree with their judgements and want to take the chance to congratulate with the authors.

Reviewers' comments:

Reviewer's Responses to Questions

**Comments to the Author**

1. If the authors have adequately addressed your comments raised in a previous round of review and you feel that this manuscript is now acceptable for publication, you may indicate that here to bypass the “Comments to the Author” section, enter your conflict of interest statement in the “Confidential to Editor” section, and submit your "Accept" recommendation.

Reviewer #1: All comments have been addressed

Reviewer #2: All comments have been addressed

**********

2. Is the manuscript technically sound, and do the data support the conclusions?

The manuscript must describe a technically sound piece of scientific research with data that supports the conclusions. Experiments must have been conducted rigorously, with appropriate controls, replication, and sample sizes. The conclusions must be drawn appropriately based on the data presented.

Reviewer #1: Yes

Reviewer #2: Yes

**********

3. Has the statistical analysis been performed appropriately and rigorously?

Reviewer #1: Yes

Reviewer #2: Yes

**********

4. Have the authors made all data underlying the findings in their manuscript fully available?

The PLOS Data policy requires authors to make all data underlying the findings described in their manuscript fully available without restriction, with rare exception (please refer to the Data Availability Statement in the manuscript PDF file). The data should be provided as part of the manuscript or its supporting information, or deposited to a public repository. For example, in addition to summary statistics, the data points behind means, medians and variance measures should be available. If there are restrictions on publicly sharing data—e.g. participant privacy or use of data from a third party—those must be specified.

Reviewer #1: Yes

Reviewer #2: Yes

**********

5. Is the manuscript presented in an intelligible fashion and written in standard English?

PLOS ONE does not copyedit accepted manuscripts, so the language in submitted articles must be clear, correct, and unambiguous. Any typographical or grammatical errors should be corrected at revision, so please note any specific errors here.

Reviewer #1: Yes

Reviewer #2: Yes

**********

6. Review Comments to the Author

Please use the space provided to explain your answers to the questions above. You may also include additional comments for the author, including concerns about dual publication, research ethics, or publication ethics. (Please upload your review as an attachment if it exceeds 20,000 characters)

Reviewer #1: The authors have satisfactorily addressed my previous comments. In particular, they have strengthened the validation of the opinion signal model by adding overlapping annotations and reporting inter-annotator agreement, added the requested information about the generation temperature, revised the references, and corrected the presentation issues identified in my review. The manuscript also now more clearly acknowledges the limitations associated with automated stance classification and the generalizability of the experimental setting. I have no further substantive concerns and recommend acceptance.

Reviewer #2: The authors have addressed all of my previous concerns thoroughly and satisfactorily. In particular, they have strengthened the theoretical framing, clarified the methodological choices, improved the presentation of the figures and analyses, and corrected the editorial issues identified in the previous version. Overall, the revisions have significantly improved the manuscript, and I believe the paper is now suitable for publication in its current form.

**********

7. PLOS authors have the option to publish the peer review history of their article (what does this mean?). If published, this will include your full peer review and any attached files.

If you choose “no”, your identity will remain anonymous but your review may still be made public.

**Do you want your identity to be public for this peer review?**  For information about this choice, including consent withdrawal, please see our Privacy Policy.

Reviewer #1: No

Reviewer #2: No

**********

# Editor report: Acceptance letter

**DOI:** 10.1371/journal.pone.0355458.r004

**Authors:** Alessandro Galeazzi

PONE-D-26-14441R1

PLOS One

Dear Dr. Coppolillo,

I'm pleased to inform you that your manuscript has been deemed suitable for publication in PLOS One. Congratulations! Your manuscript is now being handed over to our production team.

At this stage, our production department will prepare your paper for publication. This includes ensuring the following:

* All references, tables, and figures are properly cited

* All relevant supporting information is included in the manuscript submission,

* There are no issues that prevent the paper from being properly typeset

You will receive further instructions from the production team, including instructions on how to review your proof when it is ready. Please keep in mind that we are working through a large volume of accepted articles, so please give us a few days to review your paper and let you know the next and final steps.

Lastly, if your institution or institutions have a press office, please let them know about your upcoming paper now to help maximize its impact. If they'll be preparing press materials, please inform our press team within the next 48 hours. Your manuscript will remain under strict press embargo until 2 pm Eastern Time on the date of publication. For more information, please contact onepress@plos.org.

You will receive an invoice from PLOS for your publication fee after your manuscript has reached the completed accept phase. If you receive an email requesting payment before acceptance or for any other service, this may be a phishing scheme. Learn how to identify phishing emails and protect your accounts at https://explore.plos.org/phishing.

If we can help with anything else, please email us at customercare@plos.org.

Thank you for submitting your work to PLOS ONE and supporting open access.

Kind regards,

PLOS ONE Editorial Office Staff

on behalf of

Dr. Alessandro Galeazzi

Academic Editor

PLOS One
